# Community pharmacists’ views on the factors influencing prescription medication side effect counselling: a qualitative interview study

**DOI:** 10.1007/s11096-026-02230-z

**Published:** 2026-10-20

**Authors:** Perrine Evrard, Mairéad Crowley, Thomas Otway, Kieran Dalton

**Affiliations:** Pharmaceutical Care Research Group, School of Pharmacy, University College Cork, Pharmacy Building, College Road, Cork, Ireland

**Keywords:** Adverse drug reaction, Community pharmacy services, Pharmacies, Pharmacists, Prescriptions, Theoretical Domains Framework

## Abstract

**Introduction:**

Community pharmacists play a crucial role in medication counselling, which includes side effect counselling (SEC), helping support informed decision-making, patients’ adherence, and medication safety. Despite its recognised importance, deficiencies in SEC provision have been reported, and limited research has investigated the behavioural determinants underpinning pharmacists’ SEC practices.

**Aim:**

This qualitative study aimed to explore community pharmacists’ views on the factors influencing prescription medication SEC.

**Method:**

Semi-structured interviews were conducted with community pharmacists in Ireland. Transcripts were analysed using conventional content analysis and directed content analysis by using the Theoretical Domains Framework (TDF). Predominant domains were determined based on the frequency of the beliefs, presence of conflicting beliefs, and perceived strengths of those beliefs.

**Results:**

From eleven interviews, six predominant TDF domains were identified in influencing SEC provision: environmental context and resources, social professional role and identity, memory, attention and decision-making, skills, beliefs about consequences, and social influences. While SEC was considered a core professional responsibility, time pressures and workload were dominant environmental barriers. Pharmacists employ cognitive heuristics to prioritise SEC for new and high-risk medications and tailor their language and information quantity based on patients’ health literacy and emotional state to balance the need to inform against the risk of hindering adherence by overwhelming patients—skills which they believed they refined with experience.

**Conclusion:**

This study provides unique insights into the behavioural determinants influencing pharmacist SEC. The findings offer a foundation for developing targeted interventions to enhance SEC in community pharmacy, ultimately helping to improve patient safety and therapeutic outcomes.

**Supplementary Information:**

The online version contains supplementary material available at https://doi.org/10.1007/s11096-026-02230-z.

## Impact statements

- Pharmacists use an implicit attentional heuristic process to decide the level of side effect counselling patients receive, as current working conditions do not facilitate comprehensive counselling for each prescription.
- The findings suggest that giving new roles to pharmacists without matching it with adequate workforce might displace core safety-related responsibilities such as side effect counselling.
- As pharmacists revealed sometimes delegating side effect counselling, appropriate training is needed for other pharmacy staff to ensure patient safety.
- Good communication and interpersonal skills are vital for side effect counselling, as pharmacists need to balance information provision based on individual characteristics (e.g. health literacy) while minimising anxiety to ensure adherence. This calls for better attention towards these soft skills in pharmacist education.

## Introduction

Patient medication counselling is a cornerstone of community pharmacy practice, with pharmacists often being the first port of call for many communities [1–4] due to their medication expertise, a role which is highly trusted by the public [5]. Medication counselling is multifaceted, encompassing information that includes treatment purpose, dosage, precautions, storage, and potential side effects (SEs) [2, 6]. The World Health Organisation previously defined SEs as “unintended effects occurring at a normal dose related to pharmacological properties” [7, 8]. They should be considered as “(i) unintended and (ii) effects from the causal capacities or invariances of a drug” [8]. Many patients consider SE information central to their understanding and adherence to treatment [9]. By performing side effect counselling (SEC), pharmacists therefore play a pivotal role in effectively balancing communication of therapeutic benefits with potential risks, fostering patient trust and compliance.

Effective SEC enhances patient awareness, enables early detection of drug-related problems, and supports safer medication use [2, 10]. Moreover, by enabling early SE detection, SEC can prevent inappropriate prescribing cascades and reduce unnecessary treatment discontinuation resulting from misinformation or anxiety, thereby contributing to improved adherence, patient satisfaction, and overall health outcomes [2, 6, 11].

Despite the importance of SEC, its provision in community pharmacies is inconsistent, with previous research identifying barriers like time pressure and workload [2, 12]. Pharmacists also perceived that patients may be unnecessarily panicked if they are counselled on serious side effects or given too much counselling [13]. However, patients frequently report dissatisfaction regarding both the quality and quantity of information received [2, 10, 14], especially regarding SEC [9, 15]. While several studies have explored patients’ expectations and experiences, little research has investigated pharmacists’ perspectives on the barriers and facilitators underpinning their SEC behaviours [9, 10]. In particular, no study used a behavioural framework to understand what shapes pharmacists’ SEC practices.

The Theoretical Domains Framework (TDF) has been developed to identify potential determinants of health-related behaviours, and consists of a list of 14 domains gathering 128 constructs from 33 behavioural theories. By identifying domains predominantly impacting a health behaviour, the TDF establishes a foundation for the development of evidence-based strategies promoting behaviour changes and better care [16, 17].

### Aim

This study aimed at exploring community pharmacists’ views on the factors influencing prescription medication SEC, through the lens of the TDF. This in-depth understanding of SEC determinants will provide a theoretically-based foundation to inform future strategies to enhance SEC delivery and ultimately improve patient care.

## Method

Study reporting was guided by the Consolidated Criteria for Reporting Qualitative Research (COREQ) checklist [18].

### Study design

One-to-one semi-structured interviews were conducted, exploring community pharmacists’ views regarding SEC for prescription medications. Semi-structured interviews allow an in-depth, nuanced exploration of participants’ experiences and perspectives, enabling flexibility in questioning and adaptation to participants’ responses—considered particularly valuable for this under-researched topic [19–21].

### Study participants and recruitment

Eligible participants were pharmacists registered with the Pharmaceutical Society of Ireland (the national pharmacist regulator) who practise in community pharmacy settings, either regularly or occasionally. Potential participants were identified within the research team’s professional network and selected through purposive sampling, ensuring a diversity in terms of gender, years of experience, role, and work setting (e.g., rural versus urban, independent versus chain pharmacy). All participants were approached by email and provided verbal and written consent prior to interviews.

As recommended by Francis et al., we defined an initial sample size *a priori*, as well as a stopping criterion [22]. For our initial sample size, we planned to interview up to ten pharmacists (i.e. eight to ten), ensuring an appropriate representation of various pharmacist characteristics. Data collection and data analysis occurred concurrently, to help identify a point at which no new major themes were being identified. At this point, it was planned to conduct two additional interviews to help confirm that the topic was adequately explored; recruitment ceased once two additional interviews were conducted with no new identified major themes (stopping criterion). We did not conduct any repeat interview.

### Context of community pharmacy practice in Ireland

In Irish community pharmacies, pharmacists work together with pharmacy technicians (who are most typically involved in dispensing prescriptions) and other staff members that may be more typically responsible for the provision of over-the-counter medications and other front-of-shop items (e.g. cosmetics, first aid products). While a pharmacist must take responsibility for the supply of all medications in a pharmacy, they must directly oversee the dispensing of prescription medications. It is not compulsory for pharmacists to have a face-to-face interaction with all patients, so they can choose to delegate counselling duties to other pharmacy staff members. Therefore, other pharmacy staff members may be involved in the provision of medication counselling (including SEC) for both prescription and non-prescription medications. Patients can request to speak directly to a pharmacist to receive advice. Each pharmacy must have a consultation area to facilitate private conversations between pharmacy staff and patients or carers when needed.

### Data collection

A semi-structured interview guide was developed by PE and KD, based on the TDF and on relevant literature [16, 17, 23]. It aimed to explore community pharmacists’ views on the factors influencing SEC among the various TDF domains (Supplementary file 1). While the term ‘side effects’ is considered colloquial and may be used less in technical texts [24], we chose to use it throughout our study and topic guide, to better reflect day-to-day patient communication and patient-centred language. In comparison, ‘adverse drug reactions’ would have been too technical and not aligned with patient-communication in clinical practice. A pilot interview was conducted with one community pharmacist, ensuring clarity and logical sequencing of questions, which was excluded from analysis (as planned *a priori*).

Interviews were conducted online via Microsoft Teams by PE, a female pharmacist (qualified for seven years) and postdoctoral researcher at University College Cork at the time of the study, who was experienced in conducting TDF-guided interviews. The interviewer knew two interviewees in a professional capacity prior to participation. Interviews were recorded, transcribed *verbatim* by Microsoft Teams, checked by MC and TO for accuracy, and thereafter de-identified. Participants had the opportunity to ask for their transcript for validation purposes.

### Data analysis

Transcripts underwent both TDF-guided directed content analysis and conventional content analysis (within TDF domains), using NVivo® Version 15 software [25]. PE, MC, and TO independently coded the first three interviews, ensuring consistency in data analysis. MC and TO then independently coded the remaining interviews with ongoing supervisory guidance from PE and KD. PE, MC, and TO independently identified predominant TDF domains influencing SEC provision, based on criteria defined by Atkins et al*.*: the frequency of the beliefs, the presence of conflicting beliefs, and perceived strengths of those beliefs [16]. Identified predominant TDF domains were then compared and agreed on. At all stages, disagreements were resolved through discussion within the research team.

Behavioural determinants among TDF domains were identified through discussion between all team members, based on interview coding.

### Reflexivity

At the time of the study, PE and KD were academia-based pharmacists with expertise in qualitative research and TDF methodology, while MC and TO were final-year pharmacy students. All research team members had some level of practical experience providing SEC to patients in community pharmacy settings, which proved valuable for interpreting study findings. Participants were not provided with findings for feedback. Regular team meetings throughout the analysis ensured transparency, analytical rigor, and reflexivity, supporting confidence that the findings represented participants’ perspectives rather than researchers’ own experiences [26].

### Ethics approval

This study was approved by the School of Pharmacy Social Research Ethics Committee, University College Cork (reference 2025–022).

## Results

### Study participants

We contacted 23 potential participants by email, of which two declined and ten did not reply. Eleven interviews were conducted between November 2025 and February 2026. The median interview length was 39 min (range 28–55 min). Participant demographic details are presented in Table 1.

**Table 1:**
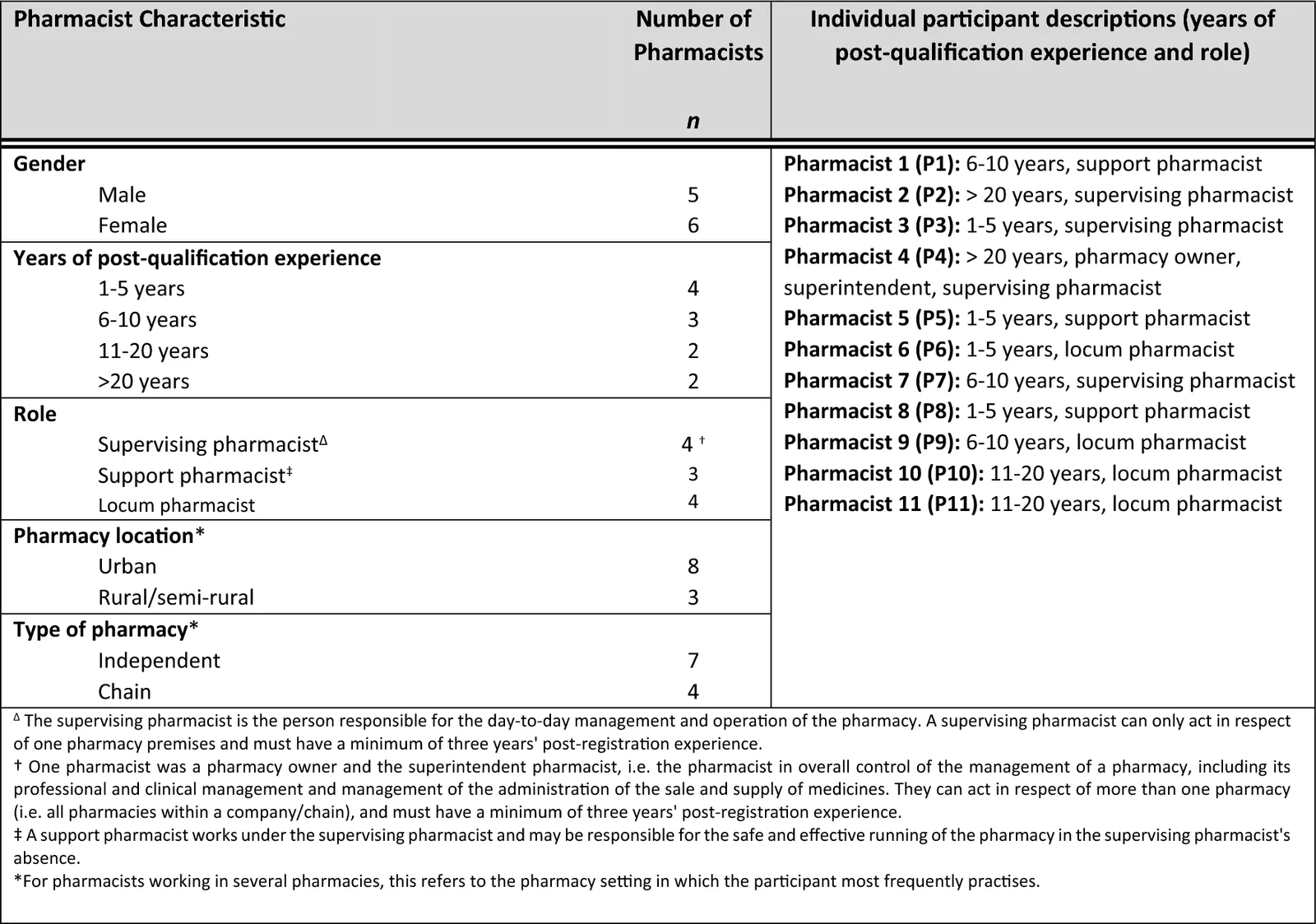
Demographic characteristics of interview participants and individual participant descriptions

### Predominant TDF domains

**Fig. 1:**
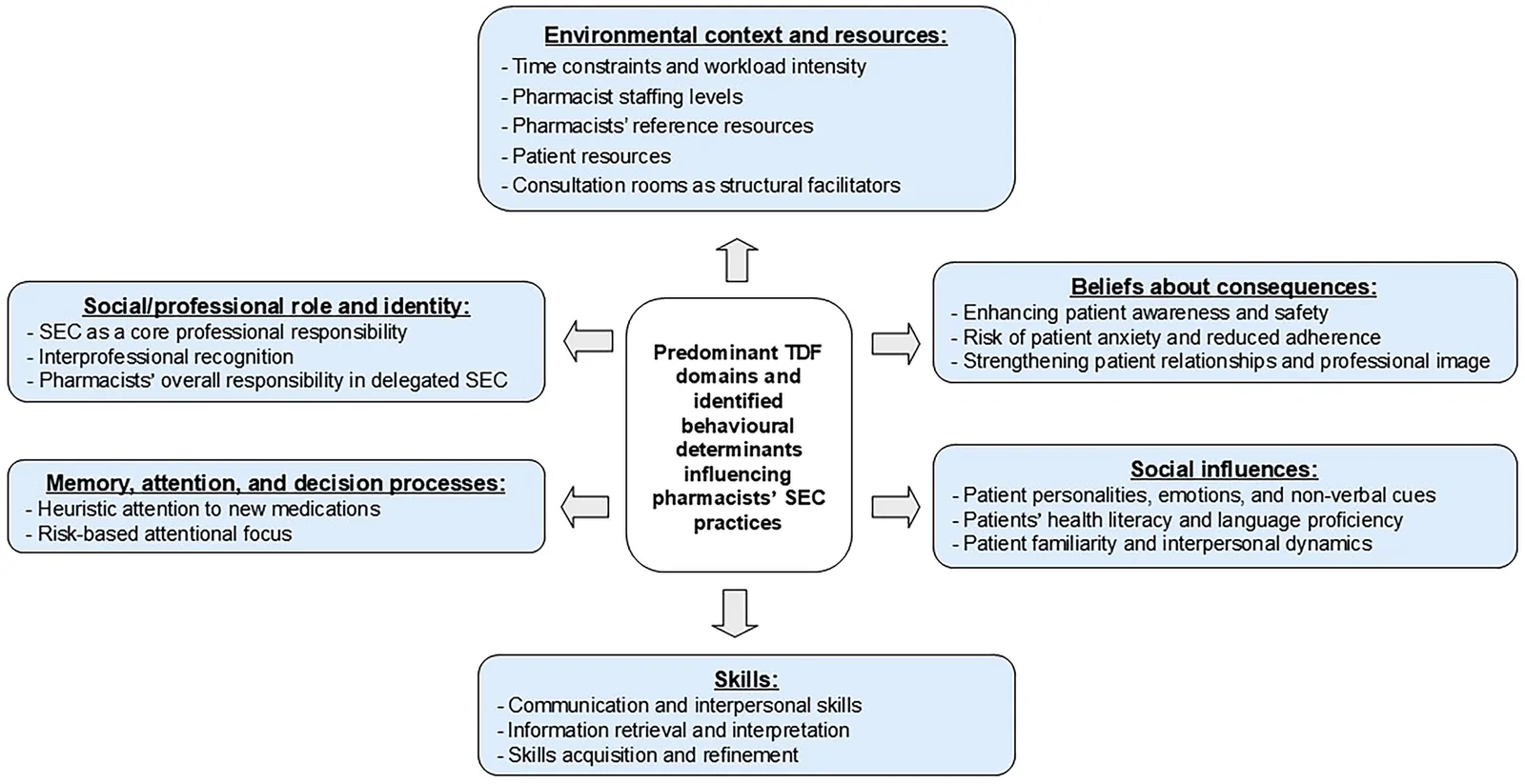
Predominant domains of the Theoretical Domains Framework and identified behavioural determinants influencing community pharmacists’ provision of SEC

While all 14 TDF domains appeared in the interview data, six were identified as predominant in influencing pharmacists’ SEC: i) environmental context and resources, ii) social professional role and identity, iii) beliefs about consequences, iv) memory, attention, and decision process, v) social influences, and vi) skills. These TDF domains are outlined in Fig. 1 and described in detail below. Supplementary file 2 contains additional quotations from these domains. Remaining non-predominant TDF domains are displayed in Table 2.

**Table 2:** Identified behavioural determinants influencing community pharmacists’ provision of SEC, aligned with the eight non-predominant domains of the Theoretical Domains Framework

Determinant	Description	Quotation
*1. Knowledge*		
Essential pharmacological knowledge	Strong pharmacological knowledge was considered important for accurate SEC. Pharmacists highlighted that understanding drug mechanisms enabled clear patient explanations. They reported knowledge gaps for new, infrequently dispensed, or specialist drugs	*“You need to know how the medication works. You need to be very familiar with the medication and the side effects that are attached to that medication.”* [P10] *“The knowledge in relation to the drug itself. […] if you have an idea of their mechanism of action you can kind of have an idea as to what side effects they can cause.”* [P5] *“There are new drugs coming on the market all the time and sometimes there’ll be a drug come across my desk that I haven’t seen before. I wouldn’t be confident in providing counselling about side effects, but at that point I would make an effort to access the SPC or to check Medicines Complete.”* [P10]
Sources and maintenance of knowledge	Pharmacists talked about the different sources of their knowledge for SEC, such as their undergraduate training, continuous professional development and daily use of resources like SPCs, as well as their own experiences in practice	*“The knowledge, yes, that’s a big part of the degree course. […] Yeah, they do a good job generally in my experience where I went to college of giving me the knowledge that I need to counsel side effects.”* [P10] *“Clinical knowledge, I totally feel like I gathered all of that knowledge through my training. I would say I didn’t acquire any of that knowledge post-training. I got all of that as part of my degree programme.”* [P8] *“But I suppose experience comes with increased knowledge as well.”* [P9]
*2. Beliefs about capabilities*		
Self-confidence enhanced by access to information resources	Overall, participants expressed high self-efficacy in delivering SEC, underpinned by ready access to digital information resources (such as the SPC and BNF). These tools enhanced their competence by facilitating rapid location, interpretation, and communication of reliable information	*“I feel confident, I know where to find the information so if a customer comes in and I don’t know the information straight away, I know I can find it quite easily and I’ll be able to understand it quite easily. I’d be confident in relaying the information I find through the SPC and the BNF.”* [P3] *“I feel confident because we have the knowledge now on the internet. If it was before the internet age and we didn’t have the SPC in front of us, it would have been very difficult. […] if the internet went down and I had to advise somebody on side effects of a medicine, it would be trickier.”* [P2]
Perceived areas for deficiency	Pharmacists were less confident with providing SEC for some specific drug classes, unfamiliar medicines, or when counselling patients with language barriers	*“I suppose the chemo [medication] would be the big one that I kind of feel a bit, you know less equipped to talk about I suppose”* [P5] *“I think it’s more for psychiatric meds where there’s more kind of interactions and stuff. I guess being able to explain the overall incidence, you know when there’s multiple different interactions going on at once. Being able to convey, you know, say the risk of those interactions”* [P6] *“One, actually well deficiency as well, I feel is that maybe if you were talking to somebody who English wasn’t their first language and depending on their level of English, it might be kind of difficult communicating with them about side effects.”* [P1]
*3. Behavioural regulation*		
Workflow prompts for consistent SEC	Organisational prompts such as ‘pharmacist consultation required’ bag labels served as environmental cues to systematically alert other staff members that patient consultation with a pharmacist was required. These self-regulation mechanisms effectively embedded SEC into routine practice	*“Putting stickers on the bag for pharmacy consultation or new medication so that the girls out the front know that ‘Oh, the pharmacist wants to talk to the person.’ […] That’s one of the practices that we do to enhance side effect counselling so that no medication goes unmissed and no patient leaves the pharmacy without talking to somebody if it’s a new medication.”* [P3] *“I will put a pharmacist consultation note on the bag so that when the patient comes in, the patient can be counselled appropriately.”* [P10]
Reflection through continuing professional development	Pharmacists maintained and enhanced their SEC skills through reflective cycles (a mandatory component of continuous professional development for pharmacists nationally), peer consultation, and structured approaches (identification of potential weaknesses and areas of improvements)	*“Continuing professional development, it’s a tool where you can reflect on parts of your practice that may require an improvement or development. It’s a useful resource when you’re reflecting on your practice to say, ‘Do you know what? I could probably structure that consultation a little better,’ and then you go through the cycle as to your reflection action.”* [P10] *“If I have this window of time that evening after work, I’ll always ask colleagues. […] I’ll always send a general text, or I actually live with pharmacists. So, I would come home and say guys, does anyone know anything about blank drug? You know what I mean? Any experiences?”* [P8]
*4. Goals*		
Proactively informing and empowering patients	Pharmacists’ overarching goals in SEC centred on informing and protecting patients to promote awareness, safety, and empowerment. They prioritised providing balanced information on medication benefits and risks, with a core objective of equipping patients with practical information in advance to support confident and effective self-management	*“It’s important that people are empowered and that they know all about their own medicines.”* [P2] *“I think it’s really important that you’re someone they can trust in terms of explaining the overall risk to them. […] I think it’s more important to explain that before it actually happens so that patients are informed, and they actually know what to do.”* [P6] *“I think it’s important for people to be aware that medicines can be dangerous and they need to be aware that it’s a balance between benefit and risk.”* [P2]
Navigating conflicting goals	Competing tasks like dispensing or vaccinations caused pharmacists to deliver less SEC or prioritise new or high-risk medications	*“…you kind of have to pick and choose like usually like new items or items that have particularly noticeable side effects or something that might be particularly dangerous to the patient or problematic.”* [P9]
*5. Intentions*		
Intentions for new medication SEC	Pharmacists expressed firm intentions to provide SEC for all new prescription medications as a routine component of daily practice	*“For a new prescription medication, I would always try to provide side effect counselling.”* [P11] *“I try to do it all the time with a new medication.”* [P3]
*6. Reinforcement*		
Patient feedback and experiences as reinforcers for adaptive SEC behaviours	Patient feedback and reactions, together with reflections on counselling interactions were key reinforcers shaping more patient-centred SEC behaviours over time. These processes encompassed both negative reinforcement (avoiding confusion) and positive reinforcement (achieving clearer communication), contingently strengthening SEC practices	*“You might phrase something in a certain way and then someone might react to that phrasing, and you might correct your phrasing. You might use a jargon word and based on the person’s reaction, you think, ‘Okay, maybe that was a bit too jargony.’ Then you might rephrase it in a certain way, and the next time you have the conversation, you’ll use the more user-friendly term.”* [P1] *“Feedback is huge on the job from patients. You may do a counselling session, and you may feel from a customer that that was too much for them or they kept asking you questions, so you didn’t give them enough information and just reflective practice and integrating, ‘How could I have done that better?’”* [P8]
*7. Optimism*		
Confidence in enhanced patient outcomes	Pharmacists expressed strong optimism that, if provided, SEC improves patient outcomes across multiple dimensions, anticipating benefits such as reduced hospitalisations and greater patient empowerment through knowledge, recognition of adverse events, and reassurance	*“I am optimistic that side effect counselling would improve patients’ outcomes in terms of adverse drug reactions, presenting to an emergency department, and serious side effects. I’m optimistic that side effect counselling done properly can pick these up before they come to a point where a patient needs to be hospitalised or worse, before it gets critical.”* [P11] *“I would be optimistic that once they’re armed with the knowledge of the side effects and what to do if they do happen [ …] I always tell people that knowledge is power.”* [P1]
*8. Emotion*		
Enjoyment and satisfaction in patient counselling	Several pharmacists described positive emotions, including enjoyment and satisfaction, stemming from patient interactions amid busy workflows, and feeling of professional fulfilment	*“I get great satisfaction from it myself because that’s what I did pharmacy for was to help people. […] I really enjoy it.”* [P5] *“I like it when I’m communicating with patients because sometimes you don’t often get to do that in a very busy pharmacy. […] it’s nice to come out and introduce yourself to the patient and have a bit of a connection with them and get to know them. So, I do like going out there, I feel happy going out there to provide the information correctly. […] it’s a positive feeling really.”* [P3] *“I have an interaction with the patient, which I always like. So that positively affects my day.”* [P8]

*BNF,* British National Formulary; *SEC,* Side effect counselling; *SPC, *Summary of product characteristics

### Predominant domain 1: environmental context and resources

#### Time constraints and workload intensity

Pharmacists identified time constraints and workload pressures as major barriers to SEC. Increasing prescription volumes and expanding clinical services (e.g. vaccinations, minor ailment schemes) were perceived to intensify workload and limit opportunities for meaningful patient interaction. Although pharmacists recognised SEC as integral to patient safety, it was sometimes seen as delaying other tasks.

*“We’re so busy […] I don’t think it’s practical for a pharmacist to be coming out too frequently counselling patients […] we’ve so many prescriptions to check, vaccinations to administer.”* [P1]

#### Pharmacist staffing levels

Adequate staffing and professional support were critical facilitators of SEC. When multiple pharmacists were present, participants felt more able to step away from the dispensary for in-depth SEC, confident that essential tasks such as prescription checking would continue seamlessly. Conversely, being the sole pharmacist on duty intensified workload and limited opportunities for patient engagement and SEC.

*“If I’m spending time out at the counter for 10 minutes talking to a patient about a newly prescribed medication, in the back of my head, I know that scripts are still being checked by the other pharmacist”* [P5]

#### Pharmacists’ reference resources

Pharmacists identified professional information resources as foundational SEC supports. The Summary of Product Characteristics (SPC) was regarded as the cornerstone of evidence-based practice, valued for its comprehensive detail on dosage, pharmacology, and SE frequency, while the British National Formulary (BNF) provided complementary guidance.

*“The SPC would be my main reference point because it has indication, dose, everything in one as a good starting point.”* [P1]

Pharmacists described a significant shift from paper-based texts to integrated digital platforms like Medicines Complete, which revolutionised the speed and depth of information retrieval.

*“Maybe 10 years ago you might reach for the BNF book. […] I find it much quicker to find a web page rather than rooting to find a book.”* [P1]

Informal online forums and patient advocacy websites (e.g. Multiple Sclerosis Society) were approached cautiously. Pharmacists valued their insight into patients’ lived experiences with specific treatments but stressed the need for critical evaluation of the information provided.

*“You have to assess the information with a bit more scepticism.”* [P9]

Some participants also envisioned future technological advancements, such as QR codes on medication labels linking to SE information videos, to further enhance patient engagement and understanding.

#### Patient resources

Patient information leaflets (PILs) were viewed as essential complements to verbal counselling. Time constraints often meant that SEC relied heavily on advising patients to read the PIL and contact the pharmacist if concerns arose.

*“You say to the person taking out the bag, ‘Tell them it’s a new medicine, the patient information leaflet is in the bag. Read it, and if they’ve any questions, call me.’.”* [P8]

While PILs were central to practice, pharmacists expressed concern that extensive SE listings could overwhelm patients, prompting them to advise cautious reading to support adherence. Pharmacists also used supplementary online resources, such as websites by the national health services provider, the Health Service Executive (HSE)—valued for their practical, patient-friendly advice on SE management. Visual educational materials were also used to communicate medication risks and benefits, though availability was limited across medications. Consequently, participants advocated for the development and dissemination of accessible, plain-language resources to support informed SEC across wider therapeutic areas.

*“The HSE resources like the warfarin and lithium leaflet are very good for giving to patients so they can have a breakdown of how to manage side effects if they do occur. I would love if they were extended to other common high-risk medications.”* [P6]

#### Consultation areas as structural facilitators

While brief explanations of straightforward SEs routinely occurred at the pharmacy counter, private consultation areas enabled more detailed and sensitive discussions. The private setting encouraged patient receptivity and inquisitiveness.

*“Patients are much less receptive to talk about their medications at the counter […] compared to going into the consultation room.”* [P7]

### Predominant domain 2: social/professional role and identity

#### SEC as a core professional responsibility

Pharmacists conceptualised SEC as a fundamental element of their professional identity and ethical responsibility as medication experts.

*“I’m fulfilling my role as a medicines expert to my patients*.” [P4]

#### Interprofessional recognition

Pharmacists believed that other healthcare professionals recognised SEC as an established and expected component of their professional role. Prescribers were perceived as supportive and appreciative, often assuming pharmacists would provide SEC.

*“It is very much seen as part of our role. If another healthcare professional […] was asked to write a job description for pharmacists, I wouldn’t be surprised if side effect counselling was part of that.”* [P8]

#### Pharmacists’ overall responsibility in delegated SEC

Clear clinical factors guided SEC delegation. Participants considered it appropriate to delegate SEC to non-pharmacist staff members for simpler medications like anti-inflammatories or antibiotics and less complex patients, while still maintaining clinical oversight. For higher-risk medications like immunosuppressants or for anxious or complicated patients, participants believed SEC should not be delegated and should be provided by pharmacists.

*“I don’t think it’s necessary for the pharmacist to deliver it all the time, but maybe it’s like in certain situations.”* [P9]

### Predominant domain 3: beliefs about consequences

#### Enhancing patient awareness and safety

SEC was perceived to enhance patient awareness, safety, compliance, and informed decision-making by empowering patients to recognise SEs, respond appropriately, and seek timely consultation if problems arose. Several pharmacists highlighted its preventative value in reducing unnecessary treatment discontinuation and prescribing cascades, noting that reassurance about common transient SEs supported patient confidence and adherence.

*“If a patient receives your counselling positively, in the event they go home and experience a side effect, they notice it as a side effect and can go back to their prescriber […] it can stop problems developing down the line.”* [P10]

Pharmacists also perceived SEC as positively impacting the healthcare system as a whole, by reducing patient hospitalisations, taking pressure off general practitioners, and saving on drug costs.

*“I do think providing counselling does reduce healthcare costs overall, number #1 by increasing compliance, you’re improving a patient’s disease state and reducing the chances of them ending up in hospital or unable to work. But also, I think you’re saving costs”* [P7]

#### Risk of patient anxiety and reduced adherence

Pharmacists acknowledged excessive emphasis or detail could unintentionally provoke anxiety, undermine confidence, or lead to treatment refusal, especially for high-risk medications. They stressed the importance of sensitive, tailored risk communication to support understanding without generating fear. To overcome this issue, pharmacists also tend to focus on the most common or clinically relevant side effects.

*“The main consequence of counselling about side effects would be that you’re making the patient a bit more anxious and reluctant to take their medicine.”* [P3]

#### Strengthening patient relationships and professional image

Pharmacists believed that SEC yielded positive relational and professional outcomes, strengthening patient trust. SEC was also perceived to enhance the profession’s public image by showcasing pharmacists’ clinical competence and patient-centred contribution.

*“It helps to cultivate the relationship with the patient a bit better. It puts you to the forefront as a healthcare professional, as knowledgeable, caring, and trying to be helpful.”* [P11]

### Predominant domain 4: memory, attention, and decision processes

#### Heuristic attention to new medications

Dispensing a new medication was perceived as a “*critical moment*” that automatically triggered SEC, reflecting an implicit attentional heuristic, whereby unfamiliar therapy warranted greater focus and patient engagement. Dose changes similarly acted as a trigger for SEC.

*“There are a couple of critical moments in your day, and when someone’s starting a new medication, that to me is one of those critical moments.”* [P7]

Conversely, repeat prescriptions rarely triggered the same attentional response. Pharmacists generally assumed that ongoing therapy signified treatment stability and sufficient patient knowledge. SEC was seldom revisited once treatment became routine, unless prompted by new safety issues, therapy changes, or patient concerns.

*“If it’s a prescription renewal, the likelihood of going out and counselling on side effects is far lower.”* [P10]

#### Risk-based attentional focus

Engagement in SEC was shaped by risk-based attentional processes. Pharmacists focus predominantly on medications associated with serious or harmful SEs (e.g., drowsiness, addiction). The depth of SEC also varied accordingly, with high-risk medications (e.g., methotrexate, anticoagulants) eliciting more detailed discussions.

*“You focus more on certain side effects from certain classes of drugs.”* [P2]

### Predominant domain 5: social influences

#### Patient personalities, emotions, and non-verbal cues

Patients’ personalities, emotional responses, and non-verbal cues influenced SEC framing and information prioritisation. Non-verbal indicators (e.g. body language, facial expressions) prompted adjustments in counselling depth and pace. Patient anxiety was particularly influential, prompting pharmacists to use reassuring language, selecting only common and clinically significant SEs, and offer follow-up support.

*“You have to take each person individually. Some people would naturally be more worried or anxious than others, you have to gauge the person in front of you. […] I might counsel one person differently than I would counsel the next person.”* [P1]

#### Patients’ health literacy and language proficiency

Patients’ health literacy, prior knowledge, and language proficiency were key influences, guiding communication strategies and prompting adaptive adjustments in SEC content and delivery.

*“Levels of health literacy is really important. Their basic understanding and how they can engage with the information.”* [P6]

Limited English language proficiency introduced additional challenges, constraining interactional depth. Pharmacists simplified explanations and employed translation tools (e.g., Google Translate) or visual aids to bridge communication gaps. Despite these strategies, language barriers frequently narrowed discussions to the most essential SEs.

*“People with different nationalities and not being able to speak English at all or very well. […] you’ll be using Google Translate. You could just be saying one or two very basic side effects.”* [P3]

#### Patient familiarity and interpersonal dynamics

Long-standing patient relationships fostered trust and understanding, enabling tailored SEC, while unfamiliarity prompted cautious, stepwise approaches to assess communication preferences.

*“Definitely it takes me longer for patients I don’t know. […] there’s a lot more questioning, and even in the information I’m giving, I am much more stepwise.”* [P8]

SEC delivery also varied with patient temperament; pharmacists offered extensive counselling to friendly, receptive patients, while providing concise, focused counselling for those perceived as argumentative. Nevertheless, participants asserted that relationship dynamics should not compromise essential safety information provision.

*“My decision will be based on the patient’s medical history, the patient’s profile, whether I ultimately believe that it’s necessary to go out and counsel on this stuff, the personal characteristics […] wouldn’t come into the equation for me.” [P10].*

#### Cultural and workplace norms

Pharmacists felt that other staff, especially the supervising pharmacist’s views regarding SEC, affected SEC provision. This highlights that different pharmacists would build different work culture, influencing other staff members they work with, and leading to disparities between community pharmacies.

*“Some pharmacists have different styles. You go into some pharmacies. I’m speaking from a locum pharmacist perspective and there will be very, very little side effect counselling going on. I think the supervising pharmacist really sets the tone for the place”* [P10]

### Predominant domain 6: skills

#### Communication and interpersonal skills

Communication and interpersonal skills were central to effective SEC, enabling pharmacists to tailor information to patients’ needs and comprehension levels, enhancing understanding and adherence while minimising anxiety. Active listening, empathy, clear language, and reassurance were essential for addressing patients’ specific concerns, eliciting relevant clinical histories, and navigating sensitive discussions to reduce the risk of non-adherence.

*“Communication skills are hugely important. […] You want to make them aware of something that is possible, but you don’t want to deter them from taking the medication.”* [P4]

#### Information retrieval and interpretation

Proficiency in accessing, identifying, and interpreting SE information effectively from reference resources was described as an essential skill. It extended beyond basic information retrieval to encompass critical data appraisal and professional judgement to distinguish information important to communicate to patients.

*“Being able to look up SPCs, to understand the adverse drug reaction section, have the knowledge of how to read it, and figure out what are the more common side effects, less common side effects.* [P3]

#### Skills acquisition and refinement

Both formal education and practical experience were deemed integral to skills development for SEC. Undergraduate training was viewed as providing a foundation in communication techniques, primarily through objective structured clinical examinations (OSCEs). However, these assessments were considered limited in replicating the complexity and unpredictability of real clinical encounters and encouraging exhaustive information delivery rather than discernment. Pharmacists deemed practical experience as central to ongoing skill refinement, as repeating patient interactions enabled them to progressively adapt their counselling techniques and language.

*“They teach you the theory of communication in college but there’s no substitute for it happening in practice.”* [P10]

## Discussion

By integrating behavioural theory with lived pharmacist experience, this qualitative study uniquely captures factors influencing prescription medication SEC. These findings establish a foundation for practice, educational, and policy initiatives to enhance SEC quality, promote behavioural change, and optimise medication safety in community pharmacy practice.

‘Environmental context and resources’ was identified as a predominant domain, illustrating how structural and organisational changes are required to enhance SEC. While SEC was regarded as integral to patient safety and professional practice, its consistent, comprehensive delivery was constrained by time pressure and workload. This tension between pharmacists’ professional identity as medication experts and time constraints, forcing them to choose which patients receive counselling from a pharmacist and how in-depth it is, has previously been reported in literature [14, 27–29]. In the context of community pharmacists’ roles expanding, including vaccination, minor ailments schemes, or sometimes prescribing, this time pressure and workload might get worse. Although role expansion is widely viewed to improve patient outcomes and alleviate system pressures, concerns have been raised that such expansion has not been matched by proportional workforce or structural supports [30, 31]. Without increased staffing or dedicated consultation time, extended services risk displacing core safety-related responsibilities, including SEC [32].

To maintain SEC quantity and quality despite competing demands, pharmacists reported delegating parts of SEC to non-pharmacist staff members like pharmacy technicians. This aligns with research highlighting technicians’ evolving contribution to patient-centred communication [33], and expressing overwhelming interest in expanding their patient-facing responsibilities, including SEC [34]. Trust was a major factor in whether pharmacists delegated SEC to pharmacy technicians as they needed to be sure in their judgment. Formal pharmacy technician training and delegation frameworks that delineate when, how, and to whom elements of SEC may be delegated would strengthen collaborative practice and support pharmacists’ workflow [35].

Pharmacists believed effective SEC would prevent abrupt treatment cessation, support early identification of SEs and management of adverse events, and strengthen therapeutic relationships. However, they also identified SEC as a potential trigger for anxiety, non-adherence, and nocebo effects if side effects were over-emphasised. Previous studies have similarly reported that concerns about frightening patients can lead pharmacists to limit the depth of counselling provided [36–38]. The present study’s participants therefore emphasised the importance of communication skills and empathy, tailoring their communication to each patient’s risk tolerance. This also explains why pharmacists reported that counselling unknown patients takes longer, as a more step-wise approach is required to assess their health literacy and level of understanding, and adjust their communication accordingly. This communication adjustment is also critical to build trust and therapeutic alliance with patients [39]. Overall, this underscores the importance of ensuring communication principles and emotional intelligence are embedded in pharmacist education.

Reliable patient reference materials were identified as key facilitators of SEC. Although PILs were valued as reinforcements of verbal counselling, their complex language and extensive, undifferentiated SE lists were viewed as potentially overwhelming for patients. This mirrors longstanding evidence that such formats can heighten patient anxiety and undermine adherence [40]. To overcome this, and similar to what has previously been reported [41], pharmacists integrated the use of digital and visual tools into their communication, including decision aids and pictographic supports. Combining visual, auditory, and textual formats has been shown to improve comprehension and recall, particularly among individuals with lower health literacy [42], representing 10–30% of adults in European Union countries [43, 44]. Future research and PILs or patient resources development should incorporate patient perspectives to ensure alignment with their informational needs. Incorporation of user-friendly language, pictographs, and clearer presentation of SE frequency and relevance should be encouraged.

This study is strengthened by its TDF use, from data collection to data analysis and results presentation. Evidence indicates that TDF-based interviews typically unearth additional insights compared to approaches lacking a theoretical basis [45, 46]. TDF use also enables the identification of behavioural determinants that would need to be targeted in future interventions to enhance SEC. Results were not presented to participants for feedback and confirmation. Still, the credibility of our findings was insured by frequent investigator triangulation and research team discussion, conducted at all stages of data analysis (i.e. transcript coding, identification of TDF predominant domains, and identification of encompassed themes). The main limitation of this work is our sampling. By recruiting via the research team’s professional network, it is possible that views may have been more homogenised due to social desirability bias [47]. While the sample reflected diversity in gender, experience, role, and pharmacy setting, this study was only conducted among Ireland-based community pharmacists, thus potentially limiting transferability of results to other healthcare settings and jurisdictions.

## Conclusion

Using a behavioural framework, this study explores community pharmacists’ perspectives on the factors influencing prescription medication SEC. These unique theory-informed insights offer a foundation for developing targeted clinical interventions and enhancing pharmacist training to improve SEC delivery in community pharmacy practice, aiming to better inform patients regarding medications’ SEs and ultimately minimise unnecessary medication-related harm in future.

## Supplementary Information

Below is the link to the electronic supplementary material.

Supplementary file1 (DOCX 63 KB)

## Data Availability

Data available from corresponding author upon reasonable request.
