# Supplementary material for: Community pharmacists’ views on the factors influencing prescription medication side effect counselling: a qualitative interview study
Source: Int J Clin Pharm. 2026 Oct 20;48(6):2525–38. doi: 10.1007/s11096-026-02230-z (PMC13731468; doi:10.1007/s11096-026-02230-z)
Supplement: Supplementary file 1 [file 11096_2026_2230_MOESM1_ESM.docx]

**Community pharmacists’ views on the factors influencing prescription medication side effect counselling: a qualitative interview study**

**Supplementary files**

Perrine Evrard^1^, Mairead Crowley^1^, Thomas Otway^1^, Kieran Dalton^1^

^1^ Pharmaceutical Care Research Group, School of Pharmacy, University College Cork, Cork, Ireland

**Journal:** International Journal of Clinical Pharmacy

**Corresponding Author:**

Dr Kieran Dalton (ORCID iD: 0000-0003-1209-8079),

Email: [kieran.dalton@ucc.ie](mailto:kieran.dalton@ucc.ie)

Postal address: Pharmacy Building, College Road, University College Cork, Cork, Ireland

Telephone number: +353 21 4901676

**Supplementary file 1: Interview Topic Guide**

We will start with two broad questions on the topic.

1. What are your thoughts on providing side effect counselling in community pharmacies?
2. In your current practice, how do you perform side effect counselling?

e.g. talk through the steps or what is done.

For the rest of the interview, I have some more specific questions about your approach to side effect counselling. Some questions might seem repetitive; however, it is important that you respond to all of them as they are derived from different theories of human behaviour and we are trying to understand which factors influence this issue.

### KNOWLEDGE AND SKILLS

3. What knowledge and skills do you think are needed to provide side effect counselling and are these part of the training you received?
4. Are there any other resources which have helped you enhance your knowledge and skills for side effects counselling, or which could help you in future?
5. Are there any areas where you have deficiencies in your knowledge or skills for side effects counselling?

- e.g. specific medication(s), specific side effect(s), approaches to mitigate side effects

### SOCIAL/PROFESSIONAL ROLE AND IDENTITY

### 6. Whose role do you think it should be to perform side effect counselling?

- Do you think it is part of your role as a pharmacist? Do you feel you are doing your job when you are performing side effect counselling?
- If they mention another staff member, ask ‘how do you decide if it is appropriate for another staff member to provide side effect counselling?’

7. Are there any pharmacist characteristics that you think are important in influencing pharmacists’ side effects counselling?

- e.g. years of experience, gender, role

### BELIEFS ABOUT CAPABILITIES

8. How confident do you feel in your ability to provide side effect counselling?

- How easy or difficult it is for you? What factors makes it easy or difficult?

### GOALS

9. How important is it to you to perform side effect counselling?
10. How does side effect counselling interfere with your usual practice?

- Are there any competing goals, including high workload, maintaining a good relationship with patients, with other healthcare professionals

### INTENTIONS

11. Think about the typical provision of a new prescription medication: how often do you try to provide side effect counselling?

- How is it different for prescription renewals?

### BELIEFS ABOUT CONSEQUENCES AND OPTIMISM

12. What do you think are the consequences of providing side effect counselling?
13. In your opinion, how optimistic are you that side effect counselling would improve patients’ outcomes?

- Explore both positive and negative consequences. Consequences can be in terms of patient outcomes, workflow, relationships with patients or with other healthcare professionals.

### REINFORCEMENT

14. Based on your previous experiences in providing side effect counselling, is there anything that now influences your decision to provide side effect counselling or your approach to providing side effect counselling?

### MEMORY, ATTENTION AND DECISION PROCESSES

15. Is side effect counselling an automatic part of your job or are there situations in which you provide more or less side effect counselling?

- Specific medications, medication classes, first prescription, known patient

### ENVIRONMENTAL CONTEXT/RESOURCES

16. What are the factors within the community pharmacy environment that act as facilitators and barriers to side effect counselling?

- e.g. workload, staffing (levels/type), consultation room or quieter area

17. Are there any resources that you use to support side effects counselling to patients?

- e.g. patient information leaflets – when do you decide to use these or not?

### SOCIAL INFLUENCES

18. What do you think are the views of other healthcare professionals on pharmacists performing side effect counselling?

- Do you feel supported in this by doctors? Do you think side effect counselling could generate conflicts?

19. How might patients or patient characteristics influence your provision of side effect counselling?

- e.g. providing counselling to a patient with lower health literacy, to a patient of the same or different gender

### EMOTION

20. How do you feel when performing side effect counselling?

- Do you ever feel worried or concerned about side effect counselling?

### BEHAVIOURAL REGULATION

21. If you wanted to implement changes in your own practice to enhance side effect counselling, what do you think would help you to do this?

That was all the questions I wanted to ask you. Is there any other important aspect of side effect counselling you can think of that we did not cover or that you would like to expand on?

| **Supplementary file 2: Identified behavioural determinants influencing community pharmacists’ provision of SEC, aligned with the six predominant domains of the Theoretical Domains Framework.** | | | |
| --- | --- | --- | --- |
| **Determinant** | | **Description** | **Quotation** |
| ***1. Environmental context and resources*** | | | |
| Time constraints and workload intensity | | Time pressure and workload intensity limited pharmacists’ capacity for comprehensive SEC, forcing continual trade-offs between optimal patient communication and workflow efficiency. | *“You have to make a judgement call...if there’s 10 scripts waiting to be checked and there’s other patients waiting outside and you’re spending 5 minutes talking to one patient about starting a new blood pressure medication, you have to decide whether that’s worth the allocation of your time.”* [P9]  *“I think things have changed a lot over the years. There was a time where we had more time to interact with customers. But the reality, I find it’s changing a lot because the busier you’re getting with vaccinations and with checking the accuracy of prescriptions you have less time to interact with customers.”* [P2] |
| Pharmacist staffing levels | | Adequate staffing enabled pharmacists to deliver comprehensive SEC, whereas being the sole pharmacist on duty sometimes limited opportunities for meaningful patient engagement. | *“It depends on the day I’m working...on Saturdays, I’d be the only pharmacist on…I have this thing in the back of my head that scripts are building up to be checked...there is definitely a time pressure when there’s only one pharmacist on.”* [P5]  *“If you know that you are the only pharmacist, you might feel a bit more under pressure, that might be a barrier to having as in depth a conversation as you would like with a person.”* [P1] |
| Pharmacists’ reference resources | | Pharmacists leveraged trustworthy resources (e.g., SPCs, BNFs) for evidence-based SEC. Furthermore, they cautiously integrated informal patient forums and anticipated future technological innovations to help optimise SEC delivery. | *“Usually, if it's a medication I don't know, I'd go with the SPC because I like that it gives frequency, I like to know how common something is, how likely it is that it will happen.”* [P4]  *“The MS Society have a really good website and people post their experiences with different treatments or medications. I would obviously stay away from unregulated forums or anything like that.”* [P8]  *“I think that technology will play a greater role […] QR codes being on the label, you scan the QR code on your phone and get a video of the pharmacist talking to you about that medicine[…] think that technology will lead to changes down the line.”* [P2] |
| Patient resources | | Pharmacists valued PILs and other accessible resources as key extensions of verbal counselling, emphasising that clear, visually engaging educational materials were essential for supporting consistent SEC and enhancing patient understanding. | *“The patient information leaflet obviously will be provided […] I would say, ‘Look, there's going to be a lot more side effects in the leaflet. You can have a little look at them […] but really don't focus too much on it because an awful lot of them would be very uncommon or very rare.”* [P4]  *“The NICE guidelines have patient decision-making aids […] It's really improved how well patients understand what the actual risk of side effects are.”* [P9]  *“If there was a printout or something for each drug […] having something that's not quite the PIL […] like a single print out with the very common, common, and red flag side effects in non-medical jargon, I think would be really handy.”* [P7] |
| Consultation areas as structural facilitators | | Private consultation areas facilitated more open and detailed SEC by providing a private setting that enhanced patient engagement and receptivity, particularly for sensitive discussions. | *“There's more privacy in that area and it can kind of appeal, I feel like patients are more able to engage in an open dialogue and ask you more about those side effects when they're in private versus if they're at a counter out in the pharmacy.”* [P6] |
| ***2. Social/professional role and identity*** | | | |
| SEC as a core professional responsibility | | Pharmacists viewed SEC as an inherent professional duty, central to their identity as medication experts. | *“Pharmacists are ultimately experts in medication, and that's something that we promote ourselves as. By definition, we should be really in a strong position to provide that information relating to the side effects of medication.”* [P10]  *“I think it's part of our professional role to do that I think we are obligated […] to make sure patients have all the information they need before they take their medication.”* [P4] |
| Interprofessional recognition | | Pharmacists’ professional identity in SEC was reinforced through interprofessional recognition of their expertise. | *“I think a lot of healthcare professionals rely on pharmacists to do side effect counselling […]. I think a lot of the time they believe that side effect counselling is part of the pharmacists’ role or mainly the pharmacists’ role perhaps.”* [P11]  *“I think they* [doctors] *feel they don't need to do it because pharmacists are going to do it, which I think is correct. I understand why they think that.”* [P8] |
| Pharmacists’ overall responsibility in delegated SEC | | Pharmacists positioned themselves as ultimately accountable for SEC, framing delegation to non-pharmacist colleagues as an extension of their professional role that allowed them to focus on complex cases while maintaining oversight, trust, and quality of care. | *“You're obviously always listening for what they're saying* [referring to non-pharmacist colleagues]*. You just need to let them know that if they're not comfortable, just come back in and ask the pharmacist. But I'm always listening to what they're saying too, to make sure there's nothing inappropriate or incorrect being said.”* [P5]  *“Facilitators is a good workflow and a knowledgeable and experienced team. If you have a team with you that you can trust to delegate some of the side effect counselling to, that’s really helpful.”* [P11] |
| ***3. Beliefs about consequences*** | | | |
| Enhancing patient awareness and safety | | SEC was viewed as empowering patients through knowledge, leading to greater awareness, safer medication use, with side effects more likely to be recognised if they appear, and to improved therapeutic outcomes. | *“If the person knows what to expect and they know how to deal with it […] they're more likely to continue with their therapy. So, if you tell a person when they start a blood pressure tablet that you might feel a little bit odd the first day or two, you might feel a bit lightheaded. That's normal […] don't panic, just give it a few days. I think if you tell them that and they are aware of that, they're more likely to continue on. […] So, I think by giving them the knowledge it empowers them to continue.”* [P1]  *“Side effects are more likely to be recognised […].it would encourage patients to perhaps return to the pharmacy or another healthcare professional if they were experiencing a side effect, if it was not tolerable and they might mention to that healthcare provider that, could this be a side effect of my medication if they were counselled appropriately about it, so that they don't end up being prescribed something else to tackle that symptom.”* [P11] |
| Risk of patient anxiety and reduced adherence | | While SEC supports informed medication use, excessive detail or emphasis risks provoking patient anxiety and reducing treatment adherence. | *“It could give them* [referring to patients] *a lot of fear around the medicine, and it could raise things that they didn't know about that medicine. And it could make them less likely to take their medicine.”* [P8]  *“If you're focusing too much on side effects, if you scare patents too much, maybe it could interfere with compliance.”* [P2] |
| Strengthening patient relationships and professional image | | SEC was perceived as a valuable means of building trust, strengthening patient relationships, and reinforcing pharmacists’ role as knowledgeable healthcare professionals. | *“Giving the patient more confidence in the pharmacist, it can be a positive thing that the patient has the knowledge that you're looking out for them or that you're letting them know what to look out for, they kind of gain increased trust in you.”* [P9]  *“I think you can have a positive impact on your relationship with your patient if you’re giving them an opportunity to discuss their medication with you.”* [P4] |
| ***4. Memory, attention, and decision processes*** | | | |
| Heuristic attention to new medications | | Pharmacists applied attentional heuristics to prioritise SEC for new medications as critical patient safety moments. In contrast, SEC for repeat prescriptions was largely omitted, guided by assumptions of patient familiarity and treatment stability. | *“If someone's starting on something new, it would be a real exception to the rule for me not to provide side effect counselling.”* [P7]  *“I would say it very rarely happens for prescription renewals. […] If there's a renewal with no change, no addition, no dose change. I would say I very rarely would do side effect counselling. I would assume, rightly or wrongly, that they have that information.”* [P8] |
| Risk-based attentional focus | | SEC was prioritised for medications perceived as high risk, directing greater attention to safety-critical drugs, while routinely deprioritising lower risk medications. | *“If it's a simple drug with a simple side effect, for example Augmentin®* [co-amoxiclav] *and diarrhoea. […] It's very much black and white. It's very much a one-line discussion, whereas if we're concerned about something that might affect your white blood cells and I'm asking somebody to watch out for a sudden sore throat or something like that. I prefer to give them, if the side effect is likely to be serious or if the side effect needs a little bit more explaining, I prefer to do it myself.”* [P4]  *“It's not as straightforward as just being able to talk to the patient about every single item that’s dispensed, it's not feasible. So, you have to pick and choose...items that have particularly noticeable side effects or something that might be particularly dangerous to the patient or problematic.”* [P9] |
| ***5. Social influences*** | | | |
| Patient personalities, emotions, and non-verbal cues | Pharmacists adjusted SEC delivery according to patients’ emotional state and non‑verbal cues, to manage anxiety, avoid information overload, and maintain trust. | | *“I would just tailor it to the patient and during the conversation you will see how well they're absorbing it. I wouldn't overload somebody if they have a lot going on or if they're struggling after the first one or two points I’m making. I would always say ‘Listen, have a little think about it. […] If you have any more questions, just give me a shout.’”* [P4]  *“If the patient is anxious or worried about taking a particular medicine […] I would take a different approach to patient side effect counselling. I would be a lot more empathetic towards the patient and provide small information so that I'm not overloading them to make them more anxious.”* [P3] |
| Patients’ health literacy and language proficiency | Patients’ health literacy, prior knowledge, and language proficiency directly influenced how pharmacists modified counselling content and depth. Lower literacy or limited English language proficiency prompted pharmacists to simplify information and use translation tools or visual aids to enhance comprehension and mitigate potential safety risks. | | *“I'd often start out with assessing what the patient knows, what they've been told by their doctor […] if they’ve been doing with their own reading first, just to see what their baseline level is, because obviously there's different levels of health literacy as well.”* [P6]    *“I think pictograms or other simpler ways of getting across side effects can be a useful tool when communicating with people who aren't as health literate or who don't have English as a native language.”*  [P11] |
| Patient familiarity and interpersonal dynamics | Established patient relationships fostered trust and more tailored SEC, whereas unfamiliar or challenging interactions prompted cautious, stepwise communication to ensure the consistent delivery of essential safety information. | | *“Over the years, I have built relationships with certain patients, some good, some bad, I suppose. The ones I have good relationships with; it's not even a question of would I go out. You automatically go out and you'd talk to them about everything. If they're not as nice, I suppose over the years you'd nearly be like ‘Oh, I don't want to talk to him or her.’ So, you keep the conversation very brief.”* [P5]  *“There can be people who are more cranky or more prone to argument. I try not to let that influence my decision to provide counselling or not […] it should be delivered regardless.”* [P9] |
| Cultural and workplace norms | Pharmacists’ delivery and frequency of delivering SEC is influenced by their peers, including by the supervising pharmacist they work with. | | *“…if they're more interested about money or, you know, selling more products and stuff like that, you kind of you'd be nearly trained to go into that kind of mode of things”* [P5]  *“It's their [the supervising pharmacist’s] personal style or whatever. Those kind of then permeate throughout the place in every area.”* [P10] |
| ***6. Skills*** | | | |
| Communication and interpersonal skills | Strong communication and interpersonal skills enabled pharmacists to deliver empathetic, individualised SEC through clear, reassuring discussions that supported understanding, confidence, and adherence. | | *“Good communication skills definitely, and good listening skills as well because a patient might have a query about one particular side effect that they might have read in a patient information leaflet. If you're not listening to their concerns, you mightn't be answering their query properly.”* [P3]  *“Communication skills or giving knowledge, but without necessarily scaring someone […] you need to translate it into user-friendly language […] it's about being calm and reassuring. So even if you're talking about the scary side effect, to reassure them that ‘look, it can happen, it's not necessarily going to happen’.”* [P1] |
| Information retrieval and interpretation | Pharmacists highlighted the importance of proficiency in sourcing, interpreting, and prioritising SE information to support safe, evidence-based SEC. | | *“The skill or the ability to quickly access information and to process it, and to deliver that to the patient […] accessing the SPC or any other relevant material quickly and accurately.”* [P9]  *“You need discernment. I think that's a really important skill […] if you look at an SPC or a PIL, there's going to be on average 50, 60 side effects reported under likely, unlikely, common. So, I think discernment is really important because you can't say all of those to a patient. I think you need to have that ability to prioritise what is important.”* [P8] |
| Skills acquisition and refinement | Pharmacists developed SEC skills through a combination of formal training and experiential learning. Practical experience was viewed as essential for refining and applying adaptive, patient-centred communication strategies in real-world practice. | | *“Very much on-the-job experience. Figuring out what best worked, how I best got my information across and what would sit best with the patient […] enough so that they're aware and would act upon any side effects, however, not enough to frighten them and deter them from taking the medication. It's a fine balance.”* [P4]  *“Experience over the years, you focus more over time on certain side effects from certain classes of drugs, I suppose that's an experience you pick up when you're getting used to dispensing the same drugs over and over again.”* [P2] |

*ADHD:* attention deficit hyperactivity disorder; *BNF:* British National Formulary; *MS:* multiple sclerosis; *NHS:* National Health Service; *NICE:* National Institute for Health and Care Excellence; *OSCE: o*bjective structured clinical examination; *PIL:* patient information leaflet; *SE:* side effect; *SEC:* side effect counselling, *SPC: S*ummary of Product Characteristics.
